# Alternative matrices in forensic toxicology: a critical review

**DOI:** 10.1007/s11419-021-00596-5

**Published:** 2021-08-19

**Authors:** Eduardo Geraldo de Campos, Bruno Ruiz Brandão da Costa, Fabiana Spineti dos Santos, Fernanda Monedeiro, Marcela Nogueira Rabelo Alves, Wilson José Ramos Santos Junior, Bruno Spinosa De Martinis

**Affiliations:** 1 Faculdade de Ciências Farmacêuticas de Ribeirão Preto, Universidade de São Paulo, Av. do Café s/n, Monte Alegre, Ribeirão Preto, São Paulo 14040-903 Brazil; 2 Faculdade de Filosofia, Ciências e Letras de Ribeirão Preto, Universidade de São Paulo, Av. Bandeirantes 3900, Ribeirão Preto, São Paulo 14040-901 Brazil; 3 Instituto Master de Ensino Presidente Antônio Carlos - IMEPAC ARAGUARI, Av. Minas Gerais 1889, Araguari, MG 38444-128 Brazil

**Keywords:** Alternative matrices, Sweat, Meconium, Breast milk, Newly emerging alternative matrices, NPS

## Abstract

**Purpose:**

The use of alternative matrices in toxicological analyses has been on the rise in clinical and forensic settings. Specimens alternative to blood and urine are useful in providing additional information regarding drug exposure and analytical benefits. The goal of this paper is to present a critical review on the most recent literature regarding the application of six common alternative matrices, i.e., oral fluid, hair, sweat, meconium, breast milk and vitreous humor in forensic toxicology.

**Methods:**

The recent literature have been searched and reviewed for the characteristics, advantages and limitations of oral fluid, hair, sweat, meconium, breast milk and vitreous humor and its applications in the analysis of traditional drugs of abuse and novel psychoactive substances (NPS).

**Results:**

This paper outlines the properties of six biological matrices that have been used in forensic analyses, as alternatives to whole blood and urine specimens. Each of this matrix has benefits in regards to sampling, extraction, detection window, typical drug levels and other aspects. However, theses matrices have also limitations such as limited incorporation of drugs (according to physical–chemical properties), impossibility to correlate the concentrations for effects, low levels of xenobiotics and ultimately the need for more sensitive analysis. For more traditional drugs of abuse (e.g., cocaine and amphetamines), there are already data available on the detection in alternative matrices. However, data on the determination of emerging drugs such as the NPS in alternative biological matrices are more limited.

**Conclusions:**

Alternative biological fluids are important specimens in forensic toxicology. These matrices have been increasingly reported over the years, and this dynamic will probably continue in the future, especially considering their inherent advantages and the possibility to be used when blood or urine are unavailable. However, one should be aware that these matrices have limitations and particular properties, and the findings obtained from the analysis of these specimens may vary according to the type of matrix. As a potential perspective in forensic toxicology, the topic of alternative matrices will be continuously explored, especially emphasizing NPS.

## Introduction

One of the goals of forensic toxicology is to apply approaches of analytical chemistry, toxicology and pharmacology for investigating compounds of forensic interest in samples collected in casework including death investigations, driving under the influence of substances, doping control, drug-facilitated crimes and more. For this reason, it is of paramount importance to investigate the presence of a given drug in biological fluids collected from the individual in a suspected-intoxication case, since this would be important evidence of drug intake. Drug testing in forensic toxicology has been historically and traditionally performed in whole blood, plasma, serum and urine specimens, which are considered conventional or traditional biological fluids [1, 2]. Since many years ago, alternative biological matrices have been explored in toxicological testing, especially due to their advantages over conventional matrices [2–4]. In general, these advantages include easier and less invasive specimen collection and in some cases, larger detection windows [3]. In addition, these matrices can be used when blood specimens are not available, degraded, potentially affected by postmortem redistribution or delayed collection after drug intake [5]. However, due to inherent characteristics and toxicokinetics, drug levels in some of these matrices may be reduced in comparison to blood or urine. For this reason, modern advancements in instrument’s technology have made possible the exploration and analysis of alternative matrices; modern and sensitive instruments are enabling the detection of lower concentrations of drugs and poisons in some of these alternative specimens [1]. Some of these samples such as oral fluid and hair are already well established and have been implemented in drug testing by several laboratories.

The goal of this paper is to discuss common alternative matrices, i.e., oral fluid, hair, sweat, meconium, breast milk and vitreous humor, considering their properties, advantages and limitations in drug testing. In addition, some of the recent studies based on the application of these matrices in the analysis of classic drugs and novel psychoactive substances (NPS) will be reviewed. Other less frequently reported biological specimens are also briefly discussed.

## Oral fluid

Oral fluid is an exocrine secretion produced mainly by the three pairs of major salivary glands (parotis, submandibularis and sublingualis) at a rate of 0.5–1.5 L per day [6, 7]. It consists mainly of water (approximately 99%), proteins, epithelial cells, bacteria, food debris and traces of drugs, composition that differs the oral fluid from the saliva [7–9].

The oral fluid is considered a direct filtering of blood because the salivary glands are highly perfused with blood [10]. The main mechanisms by which drugs pass from blood into the oral fluid are the passive diffusion (hydrophobic compounds) and ultrafiltration (low molecular hydrophilic substances) [11]. Drugs are usually present in their free fraction form since the bounded drug may not infiltrate through the salivary tissues [9]. Additionally, because oral fluid is slightly acidic relative to blood (pH 5.8–6.8), weak basic drugs tends to be ionized and, consequently, to be present in higher concentrations in this matrix (referred as ion trapping) [12, 13]. It is important to mention that many psychoactive drugs have such a characteristic, including cocaine and amphetamines [13].

The interest in using oral fluid for forensic and toxicological purposes has grown significantly in the recent years due to the advantages of this matrix, and also due to the improvement in extraction and analysis procedures. In this sense, as compared to the conventional matrices, the collection of oral fluid is simpler, easier, safer (both for patients and collection staff), painless (noninvasive) and avoids privacy issues [14, 15]. For instance, blood sampling is invasive and requires a trained health professional, while urine collection in many cases demands supervision to avoid sample adulteration [16, 17]. Both issues are overcome when sampling oral fluid. In addition, this matrix is simpler, presenting less interference as compared to blood and urine; consequently, the drug analysis can be performed more accurately [14–16, 18, 19]. Finally, oral fluid is ideal for drug online monitoring, as the drug levels in this biological matrix are thought to reflect the free drug plasma concentration, which may also reflects the drug activity [9].

Regarding of disadvantages of this matrix, oral fluid composition is influenced by several factors, such as the circadian rhythm, healthy status, age, gender, therapeutics, diet, and smoking habits [6, 9, 20]. With this respect, the interpretation of the analysis of drugs that are inhaled or smoked (such as cocaine, nicotine and heroin) may be impaired due to oral cavity contamination [9]. Additionally, the oral fluid volume for testing is limited (about 1 mL), and the analytes may be present in very low amounts, requiring very sensitive detection methods. However, the improvement in sample preparation methods (such as microextraction techniques), and the advances of analysis procedures (including gas chromatography coupled to mass spectrometry (GC–MS) or liquid chromatography coupled to mass spectrometry (LC–MS)) are efficient in reducing the impact of the last issues [8]. Finally, caution must be taken with the choice of the oral fluid sampling device, since it may directly influence the analytical result, and it is a very important variable when standardizing a method and when comparing the results of different laboratories [15]. For detailed description of the oral fluid collection devices, refer to [15, 20, 21].

For all the advantages over conventional matrices cited previously, the oral fluid is currently the most suitable alternative matrix for the assessment of recent exposure of psychoactive drugs [12]. One of the main applications is the oral fluid analysis of individuals suspected of driving under the influence of drugs, where this matrix is usually screened for cocaine, cannabinoids, amphetamines, benzodiazepines, opiates and ethanol [12, 22–26]. With this respect, oral fluid is a good alternative to breathalyzer assessment, because it provides simultaneous identification of alcohol and other psychoactive drugs, and also allows the sample to be stored and reanalyzed if there is a judicial request [16].

Another application of oral fluid is the on-site (real time) monitoring for doping purposes, as Bessonneau et al. [27] collected oral fluid to monitor in vivo 49 prohibited substances in sports, including psychoactive drugs, such as cannabidiol (CBD) and cannabinol (CBN), heroin, methadone, fentanyl, and strychnine. In addition, there are several studies that use this matrix for the analysis of drugs of abuse for clinical and forensic purposes, such as cannabinoids [28, 29], amphetamines [30, 31], cocaine [32, 33], opioids [34, 35], and benzodiazepines [36]. Finally, the analysis and identification of NPS is a hot topic in Forensic Toxicology, and oral fluid is one of the main matrices used for the assessment of these substances. With this regard, the recent studies are usually focused in analyze different classes of NPS using small volumes of oral fluid [37–39].

## Hair

Hair is a filamentous structure constituted of keratin (65–95%), water (15–35%), lipids (1–9%) and some minerals (0.25–0.95%). The average hair growth rate is about 0.35 mm per day or 1–1.5 cm per month, depending on anatomical region, ethnic origin, gender and age. When the scalp hair is not sufficient or absent to perform an analysis, the hair can be collected from other anatomical regions, such as the pubis, arms, armpits or face (beard) [3].

Hair analysis can be used to determine the concentration of many licit or illicit drugs in their parent or metabolized form such as nicotine, the biomarker of tobacco exposure [40–42]; ethyl glucuronides (EtG) and total fatty acid ethyl esters (FAEEs), which are biomarkers of ethanol intake [43–46]; cannabis [47–50]; cocaine [51–54]; amphetamines [55–57]; and NPS, including phenethylamines, piperazines, synthetic cathinones and synthetic cannabinoids [58, 59].

The accurate mechanisms involved in the incorporation of drugs into hair are still unclear. The most accepted model assumes that drugs and their metabolites are incorporated in hair by passive diffusion through blood capillaries to the cells of the growing matrix, at the base of the hair follicle. As the cells elongate and age, they die and coalesce, forming the hair fiber carrying the drug incorporated in the matrix. Other possible mechanisms are diffusion from sweat (sweat glands) or sebum to the hair as well as environmental contamination (smoke, dust or physical transfer from contaminated hands) [60].

The deposition of drugs in hair can be influenced by hair’s melanin content (hair color) and ethnic origin; and lipophilicity, polarity and basicity of the parent drug or its metabolites. Generally, dark pigmented hair tends to bind to greater amounts of drug than less pigmented hair, because the analytes are believed to bind more efficiently to the melanin found in colored hair [61]. Melanin has a hydrophobic and acidic nature, which makes this pigment responsible for the affinity of hair to alkaline drugs such as cocaine, codeine and ketamine. Lipophilic and basic molecules are more incorporated than polar analytes [60, 62]. Other parameters that can change the concentration of drugs in the hair are the difference of hair growth rate among various anatomic body sites, such as head, pubic, axillary, face and chest hair [63], the washing procedure used before hair analysis and the use of cosmetic and heat hair treatments. The products used for bleaching, perming, dyeing or relaxation contain strong bases, which may affect the stability or the amount of drug present in the hair matrix. These treatments, associated with continuous exposure to natural factors such as sunlight, weather and pollution can contribute to increase the damage in the hair cuticle [64]. In particular, photodegradation of drugs through the formation of free radicals or photosensitization reactions by intermolecular energy transfer can occur when the hair is exposed to sunlight or artificial light for many hours per day [65].

The advantages of hair analysis over other conventional biological samples are: easy and noninvasive collection (it does not require any specialized training neither violate individual’s privacy); easy transportation and storage (a solid and durable structure assure long stability); negligible risk of infection; and assessment of retrospective and cumulative drug exposure from months to years (since drugs incorporating into hair have a large window of detection). Furthermore, it is possible to evaluate chronic drug use through segmental analysis, in which hair is cut into smaller pieces and analyzed separately [66–68], and also to locate and characterize drugs on a single hair sample, using matrix-assisted laser desorption/ionization (MALDI) combined with imaging mass spectrometry [69, 70]. In postmortem cases, when investigating the presence of drugs consumed by the deceased a long time after death, a hair sample might be the only biological matrix available for the analysis.

Hair testing also has some limitations. The detection of recent drug use (within 7 days) is not possible. Accurate and sensitive methods are necessary to detect very low drug concentrations and the cost of analysis is higher than that of other biological samples. Immunoassay tests alone do not provide reliable results and the use of a confirmatory technique such as GC–MS or LC–MS [62] is needed. Interpretation of analytical findings is more complex, considering the complexity and variability of the incorporation of the drugs into hair matrix [71]. There is no consensus on “the most accurate and sensitive” method for hair analysis (washing, extraction and identification steps), which can lead to misinterpretation of very low drug concentrations. Considering this issue, the Society for Hair Testing (SOHT) provides guidelines that regulate information about collection, testing, cut-offs and reporting of confirmed results [72].

Hair analysis is of great interest in forensic, clinical and analytical sciences and the most common applications in routine analysis include workplace drug testing, drug-facilitated crime, child custody, in utero drug exposure, monitoring abstinence to drugs and/or ethanol and others [71, 73]. Therefore, hair is an alternative biological sample of large importance in forensic toxicology that allows an evaluation of historic drug use/exposure, depending on the hair length.

## Sweat

Sweat is the fluid produced by the eccrine and apocrine sweat glands. When sweat is generated on the surface of the epidermis, it evaporates slowly, fulfilling its role in the maintenance of body temperature. This fluid is mainly composed by water (around 99%) and electrolytes, carbohydrates, amino acids, urea, lactate and other organic compounds. Sweat has low tonicity and a slightly acidic nature, displaying mean pH of 6.3. However, factors such as gender, age and physical exercise, may alter this value (which can range from 5.2 to 6.9). It is believed that the volume of sweat perspired daily by the whole body of an individual can amount to between 300 and 700 mL, under normal environmental conditions [74–76]. Depending on how sweat sample is collected, it can also include secretions generated by the sebaceous glands. While hydrophilic composition represents the largest portion of this fluid, hydrophobic content may consist of less than 1% of sweat fraction, being mainly derived from sebum and apocrine secretion [77]. Within the organic portion of sweat, there are endogenous and exogenous substances excreted by the organism [78]. Mechanisms of transportation of substances, such as drugs, to sweat remain not fully elucidated. However, it is believed that the primary mechanism involved in the delivery of substances from bloodstream to sweat occurs through passive diffusion, from adjacent capillaries to the sweat glands. Substances could also reach skin’s surface by migrating through skin layers (transdermal migration). Factors governing the excretion of substances into sweat are lipophilicity, molecular mass, protein binding and pKa of the molecule [79].

A series of advantages lead us to the use of sweat as biological matrix for the investigation of compounds of interest. Sweat collection is noninvasive and can be performed in simple and safe manner. In addition, samples present less complex composition, are easier to be handled and offer less risks of pathogen transmission. Additionally, sweat samples can represent a cumulative record of substances excreted by an individual within a given timeframe. This fluid occasionally provides a greater window of detection for the identification of substances in comparison to blood: xenobiotics can be detected in sweat up to 14 days after exposure [80]. Some of the disadvantages include the rather low concentration of analytes, demanding the use of more sensitive analytical techniques to enable proper determination of targets in this matrix. In addition, there is a lack of information regarding the possibility of environmental contamination and reabsorption of substances by skin [81]. Volume of collected sample is also dependent on both internal (inter- and intra-variability in sweat production) and external factors (physical exercise, room temperature, etc.) [79].

Sweat can be collected by wiping the skin with gauze, cotton, filter paper or plastic materials [82]. For specialized collection of sweat there are proper devices available commercially, named PharmChek^®^ patches (PharmChem, Inc, Fort Worth, TX, USA). Basically, these devices consist of an absorbent cellulose patch (where the substances are deposited), which is adhered to the skin by an adjacent adhesive. The device is hypoallergenic and waterproof; besides that, each device has an imprinted individual code and once removed cannot be re-attached to the skin—such characteristics aid the maintenance of chain of custody; the latter refers to the chronological documentation of the sample from the time of collection until the receivement by the laboratory [83], recording the sequence of custody, control, analysis and disposal of materials such as biological samples, used in analyses of forensic interest that prevent sample tampering [84]. The adhesive polyurethane layer protects the patch from external contaminations, while allows gas exchanges (CO_2_, O_2_, water vapor) with the environment to occur, preventing the skin from being harmed. In this way, the device can stay attached to the skin up to 2 weeks [79, 81]. Prior application, the skin is usually cleaned with a swab soaked in 70% isopropanol, to remove previously existing substances from external environment [85]. Although being less common for drug monitoring applications, Macroduct^®^ Sweat Analysis (ELITECH Wescor^®^ Inc., South Logan, UT, USA) is another device dedicated to the collection of sweat. The system comprises a sweat inducer and collector, being originally designed for cystic fibrosis diagnosis through measurement of chloride ions in sweat. The device is composed of a 29 mm diameter disk equipped with a capillary plastic coil, responsible for the collection of fluid. Sweating induction is achieved by pilocarpine iontophoresis. Generally, the device in placed on subject’s forearm and 60 µL of sample can be obtained within 30 min. Since iontophoresis is based on the application of electrical current to the skin, the process may be uncomfortable. On the other hand, this method allows prompt collection of sweat and standard sample volumes can be used for the following analysis [77].

Parent drugs—which can more easily cross physiological barriers—are expected to be detected in the sweat at greater concentrations than their corresponding hydrophilic metabolites. In addition, because sweat pH is more acidic than blood, basic drugs tend to accumulate in this fluid rather than acid substances [78]. Sweat volume normalization remains as a pending issue. Most of the existing approaches involves the use of sodium and potassium ions as internal reference for determination of sweating rate [86, 87]. Sweat sample preparation for detection of drugs commonly involves a liquid-liquid extraction (LLE), using aqueous phosphate buffer or organic solvents (e.g., methanol). Then, the preliminary extract is subjected to clean-up to promote analyte pre-concentration. In this step, solid-phase extraction (SPE) is frequently used. Sweat analysis is generally carried out using GC–MS or LC–MS. However, direct immunoassays such as radioimmune analysis and enzyme-linked immunosorbent assay (ELISA) can also be performed in this matrix [76], although confirmatory analysis relying on traditional techniques may be required. On-site sweat testing can be performed using Drugwipe^®^ (Securetec Detektions-Systeme AG, Neubiberg, Germany). This is a pen-size device which allows to screen controlled substances such as cocaine, opiates, cannabinoids, benzodiazepines and amphetamines/methamphetamines in saliva and skin/sweat. The system has a collector site, which transfers the sample to strips containing drug-specific antibodies by lateral flow. Once the test strip is immersed in water, results can be obtained within 3–8 min. This device has been mainly used by law enforcement for roadside screening for driving under the influence of drugs [88].

Sweat analysis has important applications in both forensic and clinical toxicology. Due to the noninvasive specimen’s collection, cumulative register of substances, wider detection window and easy storage, sweat testing has been applied in the context of criminal justice system, to verify drug intake by subjects in parole/probation programs. Sweat can also be tested for drug monitoring in psychiatric outpatient service and recovering drug addicts. Additionally, sweat analysis can be used to evaluate occupational exposure to substances in the workplace, as well as to assess worker’s exposure to prescribed substances [80, 89].

Several drugs of abuse have been determined in sweat specimens, such as cocaine, amphetamines and cannabinoids [3, 78]. However, the literature on NPS testing in sweat specimens remains scarcely explored. A study on sweat analysis followed by a controlled oral administration of methamphetamine, showed that this drug was available in the matrix 2 h after dosing [90]. Doses at low and high concentrations implied in average concentrations of 63 and 307 ng/patch for methamphetamine, and 15 and 53.8 ng/patch for amphetamine [90]. Another study involving the controlled intake of 3,4-methylenedioxymethamphetamine (MDMA) indicated that the parent drug was the main detected analyte, positive in 59.7% of the samples, at < 3007 ng/patch. 3,4-methylenedioxyamphetamine (MDA) was found in 29.4% of the samples, at < 172 ng/patch [91]. A review on amphetamines and methylenedioxy derivatives showed that limits of quantitation for these analytes ranged from 1.4 to 5 ng/patch. Considering that a cut-off of 25 ng/patch is required to confirm the presence of such substances, the abovementioned methodologies seem to be suitable for sweat analysis in forensic practice [92].

Cocaine and codeine were the primary analytes found in sweat, after oral administration [82]. Peak concentrations were detected 4.5–24 h after drug intake [82]. Both analytes were identified 48 h after dosing. Concentrations of cocaine and codeine at elimination peak ranged from 33 to 3579 ng/patch and from 11 to 1123 ng/patch—for sweat collected from hand; and 22–1463 ng/patch and 12–360 ng/patch—for sweat collected from torso [82]. The developed method presented limits of quantitation ranging from 1.25 to 2.5 ng/patch, for all analytes [82]. Use of crack among drug users was also evaluated using hand fast patches; cocaine was found in 92% of samples, comparable to a rate of 91% of positive cases according to immunoassay urinalysis. In 54% of the samples, crack metabolites could also be identified, and anhydroecgonine methyl ester was the substance that was most present in the sample [93].

Paired analysis of cannabinoids was performed on hair, urine and sweat [94]. As results, Δ^9^-tetrahydrocannabinol (THC) and 11-nor-9-carboxy-THC (THC-COOH) were found in all urine and hair samples [94]. THC was detected in all sweat samples, with concentrations ranging from 0.4 to 2.0 ng/patch. Still regarding sweat, cannabinol (0.4–0.5 ng/patch) was found in 50% of cases and CBD (0.4–0.6 ng/patch) detected in 25% of samples, while THC-COOH could not be assessed in this matrix [95]. After oral administration of THC (14.8 mg/day), no daily or weekly patches presented concentrations superior to the limit of detection (0.4 ng/patch), suggesting that sweat analysis is not the most sensitive approach to investigate THC exposure [94].

Cocaine and heroin were the main analytes detected in sweat after administration of these substances by intranasal and intravenous routes [96]. Lower concentrations of ecgonine methyl ester and benzoylecgonine were also detected after 2–48 h and 8 h from cocaine administration, respectively [96]. The metabolite 6-monoacetylmorphine (6-MAM) was rapidly detected after heroin administration and its concentration continued to increase with time as heroin levels decreased, indicating heroin hydrolysis in the collection patch [96]. Estimated limit of quantitation for heroin and metabolites was 2.5 ng/patch [96]. Among drug users, heroin and 6-MAM were found in concentrations up to 400 and 441.1 ng/patch, respectively [96]. In another study, heroin and its metabolites were confirmed in 78.1% of sweat samples using GC–MS. The same samples were tested by ELISA analysis and correspondence was found in 90.6% of cases. Comparing ELISA analysis in sweat with enzyme multiplied immunoassay technique (EMIT) urinalysis, calculated sensitivity and specificity for sweat results on opiates were 68.6 and 86.1%, respectively [97].

## Meconium

Meconium is the first stool excreted by newborn. More than 98% of term infants pass their meconium within 48 h after birth [98]. However, delayed passage of meconium may occur in preterm infants and may cause intestinal obstruction [99, 100]. Other uncommon situation is the meconium passage in utero as a signal of fetal stress (hypoxia) or a signal of advanced gastrointestinal maturation (postterm infant) [101].

Unlike feces, meconium is characterized as thick, sticky, greenish-black in color and lack of odor usually inherent to regular feces [100, 102]. It is a highly complex matrix consisting mainly of water (70–75% of total wet weight) with additional components such as lipids, plasma proteins, tissue debris, enzymes, ions, hemoglobin metabolites (bilirubin and porphyrins), steroids, bile acids, sterols and mucus glycoproteins. The contents of meconium are derived from swallowed amniotic fluid, secretions of the fetal alimentary tract (bile, pancreatic and intestinal secretions) and desquamation of cells from mouth, skin, alimentary tract, vernix and lanugo hair [103]. Illicit and licit drugs, used by mother during pregnancy, cross the placenta mainly by passive diffusion and, then, are accumulated in meconium by deposition via bile and the swallowed amniotic fluid [104]. Because meconium begins to form around 11–12th week of gestation (when a fetus begins swallowing amniotic fluid) and accumulates thereafter until birth [102, 105], it has been used as alternative matrix for assessing prenatal exposure to drugs (theoretically along the second and third trimesters of pregnancy) [102, 106, 107]. In general, the frequent and chronic exposure to drugs, especially during third trimester of pregnancy, are required to produce positive results for drug(s) in meconium [108]. Different patterns or proportional concentrations of the drug or metabolite in meconium may occur among newborns with similar exposure and, even between dizygotic twins. The mismatches may be explained by differences in fetal metabolism and placental differences [109, 110].

The main advantage of meconium as biological matrix is its wide detection window of in utero drug use. Others advantages of meconium include: noninvasive and easy sampling (meconium is collected from the diapers); a large amount of sample is available for collection (total amount range 20–60 g), and a small amount of sample is required for analysis (less than 1 g) [102, 111]. On the other hand, meconium testing has some limitations. It does not provide information about fetal drug exposure in the first trimester of pregnancy; meconium can be contaminated by urine or traditional feces (milk stool); usually it is not readily available for collection; meconium may be lost if is excreted in utero; it is not an homogenous sample (inhomogeneous distribution of drugs in meconium); irregular accumulation of meconium in the fetal gut (nearly 1 g of meconium accumulates until 23–26 weeks of pregnancy, 5 g until 27–32 weeks and 80% of meconium accumulates after 38 weeks); drugs administered during labor and delivery may be detect as well as drugs administered to newborn before meconium collection [111–114].

Drugs may be screened in meconium using immunoassay techniques (e.g. ELISA, EMIT, biochip microarray) [115], then confirmed and quantified using GC–MS or liquid chromatography–tandem mass spectrometry (LC–MS/MS) techniques [113]. As all complex matrices, meconium requires extensive pre-analytical processing to minimize matrix interferences and improve the detection potential for the analytes of interest. These processing involves sample homogenization (with water or organic solvents) and further extraction using, mainly, LLE followed by SPE, accelerated solvent extraction or headspace solid-phase microextraction (SPME) [114, 116].

Several licit and illicit drugs of forensic interest have been determined in meconium specimens. Assessment of fetal alcohol exposure can be performed via quantification of non-oxidative metabolites in meconium: EtG or FAEEs, represented by 9 compounds. Unlike EtG, FAEEs from mother do not across the placenta and thus FAEEs in meconium are produced by the fetus from ethanol that crossed the placenta, reflecting the true fetal alcohol exposure [117]. On the other hand, EtG in meconium is more stable at room temperature and its levels in meconium have been better correlated with EtG levels in maternal hair and with maternal self-report than FAEEs in meconium [118]. Himes et al. [119] concluded that maternal alcohol consumption at 19 weeks or more was better represented by meconium EtG levels equal to or higher than 30 ng/g rather than currently used FAEE cut-offs.

Others biomarkers in meconium have been determined for identification of fetal exposure to tobacco (nicotine and cotinine); cocaine, hydroxybenzoylecgonine, benzoylecgonine, ecgonine methyl ester and anhydroecgonine methyl ester); cannabis (THC-COOH); amphetamine, *p*-hydroxyamphetamine; methamphetamine *p*-hydroxymethamphetamine); heroin (6-MAM, morphine and codeine) [107, 108, 110]. In regards to NPS, in the literature, there are only a few reports on analytical method development and detection in authentic specimens (e.g., synthetic cathinones [120–122]).

Meconium drug testing has some limitations that should be considered in the interpretation of the results. A false negative result may be due to instability of some analytes in meconium (e.g., FAEEs, 6-MAM); inappropriately high cut-off concentration; delayed meconium collection (collected sample is transition stool or milk stool); inappropriate analytical targets (analytes not common in urine can improve detection of drug in meconium, e.g., *m*-hydroxybenzoylecgonine and *p*-hydroxymethamphetamine) [110, 123]. A false positive result for FAEEs may be due to delayed meconium collection because of contamination of meconium with dietary components of stool produced after birth and ethanol-producing microorganisms. Then, in this case, there is a recommendation that meconium collection should be performed until 24 h after birth [124]. Despite these limitations, meconium drug testing remains the “gold standard” for identifying in utero drug exposure (drug exposure detection in newborn). More studies are needed to associate meconium drug concentration with the degree of exposure or the severity of outcomes [114].

## Breast milk

Human breast milk (HBM) is a complex fluid with high lipoprotein content, and is considered as the best nutrient for newborns aging from 6 months to 2 years old and also works as a complement to solid food [125–127]. Several exogenous compounds arising from mothers’ consumption habits may be incorporated into breast milk during lactation, and breastfed babies may be exposed to the substances such as medication, pesticides, toxic metals and drugs of abuse [128–131]. In forensic toxicology, HBM is an alternative matrix for drug analysis with a short detection window, being useful to investigate mother’s recent drug use (hours) and to assess the infant exposure to substances that may be harmful for their cognitive and motor development [132, 133]. It is also noteworthy that the noninvasive and easy collection of this sample and the importance of the analytical results complement the information from self-reports [134, 135]. The benefits associated to breastfeeding for both mother and infant, including decreased rates of infections and severe comorbidities, must be weighed against the effects of the drug on the infant to make the best decision for both mother and child’s health.

Advances in analytical chemistry provided new techniques that offer adequate sensitivity, environmental friendliness and ability to fast process many samples of different complexities [136]. In a recent review about HBM and psychoactive substances by Santos and De Martinis [137], most studies published over the last 10 years reported analysis of HBM using LC–MS/MS, and the most commonly employed sample preparation techniques were SPE, protein precipitation, LLE and some miniaturized techniques. More detailed information is summarized and published elsewhere [137]. More recently, Behpour et al. [138] developed a gel-based electromembrane microextraction (EME) coupled with switchable hydrophilicity solvent-based liquid–liquid microextraction procedure to quantify antidepressants in breast milk, serum and wastewater samples. The technique provided growth of enrichment factor over original EME, reducing limits of detection (LODs) and limits of quantification (LOQs); and the use of low volume of organic solvent allowed the analysis by gas chromatography with flame ionization detector (GC–FID), which usually cannot be performed with EME due to its aqueous acceptor phase, which is often analyzed by high-performance liquid chromatography with ultraviolet detection (HPLC–UV) [138]. Sempio et al. [139] developed an LC–MS/MS method for quantification of 12 cannabinoids in 30 samples from a clinical study and 6 samples from a breast milk bank and compared the analysis’ results with ELISA immunological assay. They performed protein precipitation followed by online extraction using 0.2 mL of sample, achieving lower LOQs than ELISA’s LODs for THC-COOH and adequate absolute recoveries [139]. Results showed that the 30 clinical samples tested negative on ELISA, but all of them tested positive for THC using LC–MS/MS, and in many of them, 11-hydroxy-Δ^9^-tetrahydrocannabinol (THC-OH), THC-COOH, CBD and cannabigerol were also detected [139]. All the 6 milk bank samples tested negative on ELISA and on LC–MS/MS as well [139]. The results of this study show the importance of correctly interpreting the results of screening tests and the need of greater implementation of quantitative analyses in the routine of toxicological assessment. The absence of THC-COOH according to ELISA would mean that there was no recent consumption of cannabis, while a highly sensitive technique showed the opposite result, suggesting the possibility of infant exposure [139].

The excretion of substances into HBM depends on physicochemical characteristics of the compound, including molecular weight, ionization degree (pKa) and the solubility in lipids as well as the pH of milk (6.5–6.8) [140]. For instance, THC, the major psychoactive compound of cannabis, has low molecular weight and high pKa value, is lipid soluble and 99% is protein bound. Those factors cause the THC transfer into breast milk and its accumulation in lipid-filled portions as well [141]. Cannabis is the most consumed drug of abuse in pregnancy and after birth, but still there is no information about how much the amount present in HBM is related to the concentration of THC in cannabis or its joints, the frequency of use and the concentration in maternal plasma [127, 142]. Two surveys evaluated the effect of maternal marijuana use while breastfeeding, and they found that infants exposed to marijuana were slightly smaller and had decreased points in motor scores, but marijuana use during pregnancy might have caused confounded results [143, 144]**.** Some studies reported cases of infant cocaine intoxication through breast milk presenting acute effects as agitation and seizures, neonatal abstinence syndrome in cases of opioid intoxication, and sedation and breast sucking difficulty due to exposure to cannabis [145, 146]. Several clinical studies about infant exposure through breastfeeding are summarized in Drugs and Lactation  Database [147].

The lack of studies shows the importance of public policies to inform population about the consequences of infants exposed to drugs of abuse through breast milk. The risk assessment in breastfed babies requires more quantitative data, as well as an understanding of the factors determining exposure after oral maternal ingestion followed by excretion into HBM. Furthermore, confirmatory toxicological analysis should be included in quality control routines of breast milk samples (complementing self-reports and screening tests) to maintain its quality and the importance of breastfeeding. In addition, combining clinical data with in silico modeling and simulation approaches (such as physiologically based pharmacokinetic model, chemometrics and others) is an emerging and powerful strategy for predicting drug mechanisms and toxic effects in human body [148].

## Vitreous humor

Vitreous humor (VH) is an alternative matrix commonly used in postmortem toxicological analyses and its application in forensic analysis started in the 1960s [149]. Since then, many analyses in VH have been conducted, including ethanol, illicit drugs and endogenous compounds [150]. One of the interests of using VH in toxicological analysis is especially when traditional matrices, such as blood or urine, are unavailable or under inappropriate conditions for analysis [151]. Casework involving embalmed, burned or highly decomposed bodies are such examples [152].

VH is a gelatinous liquid filling the eyeball, between the crystalline lens and the retina [2, 149][143]. The composition of VH is primarily water (around 98%), including other components such as lipids, electrolytes, polysaccharides, proteins and other substances [149, 151]. Drugs present in circulating blood may reach the VH through the blood-retinal barrier, by passive diffusion or active transport [153, 154]. Drugs are able to reach the VH only if in free form (not bound to proteins) [154], and thus drugs exhibiting a low rate of protein binding are present in VH at greater levels [154, 155]. On the other hand, if a drug is likely to bind to proteins, it should not be expected in VH [154].

The application of VH in forensic analyses is based on several advantages of this matrix. In comparison to other postmortem fluids, the composition of VH makes it a cleaner matrix, with less interferents [2, 153], being an aqueous matrix with minimal protein contents [151]. VH also has a low number of cells and lacks of blood vessels [153], avoiding potential infections from the blood [155]. Another advantage of VH is its prolonged stability in comparison to other matrices [2, 156]. The location of VH is also an advantage, as it is located in a body compartment protected against contamination and microbial activity [149, 151], especially being remotely located from gastrointestinal tract [150]. In comparison, postmortem blood is more likely than VH to suffer microbial activity and degradation [157] and autolytic processes are delayed in VH in relation to blood [158]. After death, bacteria present in the gut, lung, oral cavity and other microbiomes, and also environmental bacteria may infect other surrounding tissues and fluids, whereas in VH this process occur to a lesser extent, reducing the contamination and increasing the stability of this specimen [149]. For example, in a study by Harper [157], postmortem blood and VH were collected from 51 decedents and microbiological analyses revealed no significant amounts of bacteria or fungi in VH and high diversity of microorganisms in 32 postmortem blood specimens. This is particularly interesting for ethanol testing, since the postmortem formation of ethanol is unlikely in VH [159]. In VH, there is no esterase activity [160], which also contributes to an increased stability of compounds in this specimen. Although the use of VH has several analytical benefits, drug testing in VH has a few limitations. The volume available for sampling is limited and the blood-retinal barrier may restricts the incorporation of drugs into VH [156, 159]. In addition, in violent death cases a rupture of the eyeball may occur, with loss of VH.

The collection of VH is performed by gentle introduction of a sterilized needle into the eyeball followed by fluid aspiration [149], penetrating in approximately 2 cm [2] and avoiding the collection of retinal or iris cells [153]. Usually sufficient volumes are collected for analysis but the volume may be reduced during postmortem body dehydration process [149]. Around 2–2.5 mL of VH is available in each eyeball [152]. Water or saline solution may be used as surrogate for VH, to provide the physical aspect of the eyeball, after VH is removed [153, 161], especially for funeral purposes. Usually samples are stored in polypropylene or glass tubes, without adding any preservatives or adding fluorides (sodium or potassium), oxalates (calcium or potassium) or ethylenediaminetetraacetic acid (EDTA) disodium salt [151]. For example, in a study by Rees et al. [160], it has been shown that sodium fluoride addition to VH provided greater stabilities for 6-MAM, which could be explained due to reduction of bacterial activity and putrefaction in this sample.

In general, VH testing in a forensic toxicology setting does not differ from blood or urine testing and several methods developed for blood/urine testing have been adopted in VH analysis [154]. As previously mentioned, the protein and aqueous contents in VH samples tend to reduce the complexity of sample preparation [151]. A systematic contemporary review covering methods used in the preparation and analysis of VH specimens has been recently published by Wójtowicz et al. [151]. In general, several extraction techniques have been explored for VH samples’ preparation. In the literature, there are several SPE-based methods available proposed for extracting drugs from VH specimens [154–156, 162–168]. LLE has also been reported and explored for VH samples [169–175]. Other techniques have been less frequently explored such as microwave assisted extraction [176], disposable pipette extraction [177], supercritical fluid extraction [178], dispersive liquid-liquid microextraction [179] and liquid-phase microextraction (LPME) [180].

A diverse repertoire of analytical techniques has been reported in the analysis of VH specimens. Chromatography coupled to mass spectrometry-based methods have been frequently used due to its high sensitivity, selectivity and accuracy [151]. Gas chromatographic techniques used in separation and analysis of VH samples include GC–MS [154, 156, 163, 164, 167–170, 180–182], gas chromatography with nitrogen-phosphorus detector (GC–NPD) [170, 177] and GC–FID [159, 166]. Gas chromatography coupled to tandem ion trap mass spectrometry (GC–MS/MS) has also been applied in the analysis of cocaine in sheep’s VH [183]. Analytical methods using LC have been developed as well, using diverse techniques such as HPLC–UV [162], high-performance liquid chromatography–diode array detection [155, 171, 176, 179, 184], LC with fluorescence detector (e.g., [174]), LC–MS/MS (e.g., [173, 175, 185]) and liquid chromatography coupled to time-of-flight mass spectrometry (LC–TOF-MS) [165]. Comprehensive screening of VH (covering a large range of compounds) and comparison to other traditional matrices (e.g. blood or urine) have been performed by GC–MS [156] or by LC–TOF-MS [165]. Separation methods based on capillary electrophoresis have also been explored for drug testing in VH such as a capillary electrophoresis with diode array detection method published by Costa et al. [172]. Besides instrumental separation methods, immunoassays applied in the analysis of other biological fluids have also been explored for VH [170, 186–189].

Over the years, VH has been successfully used in forensic casework, for illicit drugs and NPS testing. Several classic drugs of abuse have been detected in authentic VH specimens such as the following examples: ethanol [159, 181, 190], cocaine and its metabolites [155, 164, 166, 172, 176, 181, 190–192], opioids and/or their metabolites [155, 167, 168, 173, 176, 177, 182, 185, 190], amphetamine and/or methamphetamine [164, 173, 190], MDA derivatives [169], ketamine [172], phencyclidine [154, 170], LSD and its metabolites [175], γ-hydroxybutyric acid [193], benzodiazepines [171, 173, 184, 190], antidepressants [173, 180, 194, 195] and barbiturates [196]. On the other hand, VH does not seem to be a good biological matrix for investigating cannabinoids [151]. In a study by Saenz et al. [197], THC and THC-OH were detected only in blood and/or urine but not in VH in two cases [197]. In these same two cases, THC-COOH was detected in blood, urine and VH in one case whereas in the other case THC-COOH was detected in blood and urine only [197]. Similarly, in another study by Peres et al. [164], THC and THC-COOH were detected in three cases in whole blood specimens but not in VH. Other studies in the literature reported findings regarding THC and its metabolite distribution into VH in agreement with these observations [170, 198]. This can be rationalized due to strong affinity for plasma proteins and lipophilicity exhibited by THC, which reduces the incorporation of THC into aqueous specimens, such as VH [164, 199]. In addition, Lin and Lin [198] observed low vitreous concentrations of THC-COOH, a hydrophobic character compound. In regard to NPS, VH has also been implemented in toxicological analysis for a different classes including fentanyl and non-fentanyl opioids [200–207], synthetic cathinones [208–212], designer benzodiazepines [190], 5-(2-aminopropyl)indole [213] and phenethylamines [214–216].

## Other alternative matrices

Oral fluid, meconium, hair, sweat, breast milk and VH are commonly reported and implemented in forensic toxicological analyses as alternative matrices. However, this range of alternative specimens is not exhaustive and other fluids have been explored and proposed. The use of other fluids and tissues including bile [5], nails [217], bone marrow [218], umbilical cord [107, 113, 219] and others, as alternative matrices has been reviewed and discussed in the literature. In addition, tooth is another matrix that has been explored in forensic toxicology, especially considering its ability to resist to postmortem decomposition, environmental changes and other agents [220]. Examples of drugs or metabolites recently reported in human teeth include cocaine, benzoylecgonine, morphine, 6-MAM, THC, CBN, CBD [220], amphetamine, MDMA, morphine, codeine, norcodeine, methadone,  2-ethylidene-1,5-dimethyl-3,3-diphenylpyrrolidine, fentanyl, tramadol, diazepam, nordiazepam and promethazine [221]. Another alternative specimen more recently explored is cerumen or earwax. Cocaine and metabolites, methamphetamine, opioids, cannabinoids, benzodiazepines, antiepileptics and antipsychotics are examples of substances recently reported in authentic cerumen specimens [222, 223]. Meier et al. [222] have also detected the NPS 4-fluoroamphetamine in cerumen specimens. The collection of cerumen is less invasive than that of other biofluids [223] and the detection window is longer when compared to urine [222]. Synovial fluid obtained from articular joints, especially knee joints, has been also proposed as an alternative matrix for the investigation of drugs/metabolites such as morphine, codeine, cocaine, 6-MAM, benzoylecgonine and ecgonine methyl ester [224].

## Conclusions

The selection of specimens in toxicological analyses is a critical step. Understanding the properties of both target analyte and matrix is very important, to select the most appropriate biological fluid/tissue for the case under investigation. Alternative biological matrices are biological fluids/tissues that can provide additional information and advantages in comparison to blood and urine testing, in several aspects such as sample collection, detection window and complexity of sample preparation/analysis. In addition, these matrices can be collected and analyzed when blood and urine are not available. However, each one of these alternative matrices has its own characteristics, advantages and limitations, which need to be considered. The perspective in forensic toxicology is that alternative matrices will be more frequently explored in the future. For example, the Organization of Scientific Area Committees for Forensic Science (OSAC) recommend research on alternative matrices for the improvement of forensic toxicology [225]. Therefore, further research is still needed to provide analytical methods and better understanding on the behavior of drugs in these matrices, especially for emerging NPS.
